# The effects of neck flexion on cerebral potentials evoked by visual, auditory and somatosensory stimuli and focal brain blood flow in related sensory cortices

**DOI:** 10.1186/1880-6805-31-31

**Published:** 2012-12-03

**Authors:** Katsuo Fujiwara, Kenji Kunita, Naoe Kiyota, Aida Mammadova, Mariko Irei

**Affiliations:** 1 Department of Human Movement and Health, Graduate School of Medical Science, Kanazawa University, 13-1 Takara-machi, Kanazawa 920-8640, Japan; 2 Department of Sports Instruction, Faculty of Sports and Human, Sapporo International University, 4-1-4-1 Kiyota, Kiyota-ku, Sapporo, 004-8602, Japan; 3 Department of Rehabilitation Science, Osaka Health Science University, 1-9-27 Temma, Kita-ku, Osaka, 530-0043, Japan

**Keywords:** Brain activation, Focal brain blood flow, Near-infrared spectroscopy, Neck flexion, Sensory evoked potential

## Abstract

**Background:**

A flexed neck posture leads to non-specific activation of the brain. Sensory evoked cerebral potentials and focal brain blood flow have been used to evaluate the activation of the sensory cortex. We investigated the effects of a flexed neck posture on the cerebral potentials evoked by visual, auditory and somatosensory stimuli and focal brain blood flow in the related sensory cortices.

**Methods:**

Twelve healthy young adults received right visual hemi-field, binaural auditory and left median nerve stimuli while sitting with the neck in a resting and flexed (20° flexion) position. Sensory evoked potentials were recorded from the right occipital region, Cz in accordance with the international 10–20 system, and 2 cm posterior from C4, during visual, auditory and somatosensory stimulations. The oxidative-hemoglobin concentration was measured in the respective sensory cortex using near-infrared spectroscopy.

**Results:**

Latencies of the late component of all sensory evoked potentials significantly shortened, and the amplitude of auditory evoked potentials increased when the neck was in a flexed position. Oxidative-hemoglobin concentrations in the left and right visual cortices were higher during visual stimulation in the flexed neck position. The left visual cortex is responsible for receiving the visual information. In addition, oxidative-hemoglobin concentrations in the bilateral auditory cortex during auditory stimulation, and in the right somatosensory cortex during somatosensory stimulation, were higher in the flexed neck position.

**Conclusions:**

Visual, auditory and somatosensory pathways were activated by neck flexion. The sensory cortices were selectively activated, reflecting the modalities in sensory projection to the cerebral cortex and inter-hemispheric connections.

## Background

Humans are capable of maintaining a variety of postures, from which the most suitable preparatory posture is selected for intended exercise. Howorth [1] has surveyed various exercise postures using motion pictures and has observed that a basic dynamic posture in which the ankle, knee, hip and neck joints, and trunk are all slightly flexed is common for different sudden initiation of motions and when pursuing a rapidly moving visual target. A flexed neck position leads to non-specific activation of the brain, resulting in shortened saccadic reaction time [2,3], increased amplitude of the late component of the contingent negative variation (event-related potential) [4], and increased amplitude and shortened latency of motor evoked potentials evoked by transcranial magnetic stimulation [5]. The non-specific activation is presumably due to ascending activation associated with muscular-sensory information from the neck extensors, and/or descending activation from the cerebral cortex, which includes attention-related processes [6-10]. In a previous study, saccadic reaction time decreased during vibration of the trapezius muscle when the neck was in a resting position [11]. This finding supports the existence of ascending brain activation from the trapezius muscle. In 1949, Moruzzi and Magoun [12] proposed that the ascending brain activation system originates at the brainstem reticular formation, and since then the system has been examined using animal studies, pharmacological experiments and neurological treatments for patients with brain dysfunction [10,13,14]. To date, the activation system is known to consist of two subsystems: a dorsal pathway from the reticular formation to the thalamus and cortex, and a ventral pathway from the reticular formation to the hypothalamus and cortex [13,14].

Visual, auditory and somatosensory information are important for perceiving the surrounding environment, the location of the whole body in the environment and the position of each segment in the body. Early shift in sensory information processing time and enhancement of activity in the sensory cortex are useful for those perceptions. Visual, auditory and somatosensory information are processed via subcortical and cortical neural pathways that have been described in detail as follows. Information processing induced by visual, auditory and somatosensory stimuli has been examined using sensory evoked potentials. With sensory evoked potentials, the effect of descending brain activation on information processing, mainly from the cerebral cortex, is relatively low compared with the effect of the event-related potential. Thus, it is probable that the sensory evoked potentials are affected by brain activation with maintaining neck flexion position. The maintenance of a flexed neck position shortens the P100 latency of visual evoked potentials (VEP) [15] and increases the amplitude of the middle-latency component of auditory evoked potentials (AEP) [16]. However, these experiments were conducted on different days and in different groups of participants. The effect of neck flexion on the somatosensory evoked potentials (SEP) has not yet been investigated. If neck flexion has effects on VEP, AEP and SEP in the same participants, it would suggest that visual, auditory and somatosensory pathways are commonly and selectively activated with neck flexion.

When studying evoked potentials it is hard to locate the area of the sensory cortex that is activated, as the potentials have a high time resolution and a low spatial resolution. To compensate for the insufficient spatial resolution, the location of the sensory cortex activity has been measured using hemodynamic recording methods [17-19]. By simultaneously recording the sensory evoked potential and the focal brain blood flow (FBBF), it is possible to identify the brain region that is activated by the evoked potential. Previous studies have investigated hemodynamic responses related to the neural activity in each sensory cortex using functional magnetic resonance imaging (fMRI) and near-infrared spectroscopy (NIRS) [17-19]. Although NIRS has lower spatial resolution than fMRI, it has the advantage of permitting examination of brain activity without body fixation and is considered to be an optimal method for measuring hemodynamics during neck flexion [20].

In this study, we investigated the effects of neck flexion on VEP, AEP, SEP and FBBF in each sensory cortex. We hypothesized that the decrease of latency and the increase of amplitude in VEP, AEP and SEP, and an increase in the blood flow in each cerebral sensory cortex would be found while maintaining the neck flexion position.

## Methods

### Participants

We previously reported that the significant shortening of saccadic reaction time associated with maintenance of neck flexion was observed in participants who belonged to a high-speed ball sports club; that is, a training effect of neck flexion on the brain activation existed [2,3,20]. In the preliminary experiment, we measured the saccadic reaction time during maintenance of neck flexion for 30 randomly selected healthy adults. Of these adults, 12 participants (two men, ten women) who showed significant shortening of the saccadic reaction time participated in this study. Mean value for age was 23.3 years (SD = 2.9). No participant reported any history of neurological or orthopedic impairment. In accordance with the Declaration of Helsinki, all participants provided informed consent by signing a consent form after receiving an explanation of the protection of privacy rights and experimental protocols, which were approved by our institutional ethics committee.

### Apparatus and data recording

#### Experimental setup

**Figure 1:**
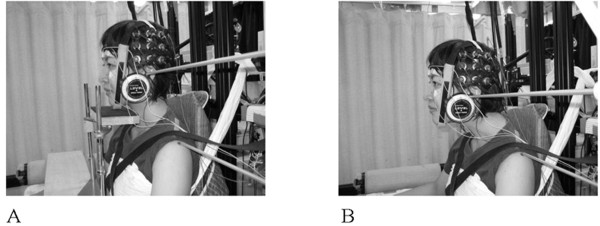
**Pictures showing experimental setup for simultaneous recordings of auditory evoked potentials and oxidative**-**hemoglobin concentration in the auditory cortex.** (**A**) Resting neck position. (**B**) Flexed neck position.

The experimental setup is shown in Figure 1. Participants sat on a steel-framed chair with their back resting against a vertical wall and their trunk secured by two acrylic belts and a cotton band to prevent anteroposterior movement. Participants kept their knees flexed at approximately 90° and rested their feet on a low table. The neck flexion angle was defined as the rotational angle of the tragus around the acromion in the sagittal plane, with the starting position (0°) being a quiet sitting posture. This flexion angle was strictly determined using a custom-made angular detector in which the center point was set at the acromion and the distance between the acromion and the tragus was regulated. The head inclination angle was determined as the angle between the auriculo-infraorbital line and the gravitational line, in order to maintain constant sensory stimulation of the vestibular organs. An angular detector (Level+angle detector; Mitsutomo, Tokyo, Japan) using the principle of a pendulum was placed on the temple to determine the head inclination angle. A chin stand was used to support the head and to allow relaxation of the neck extensor muscles as much as possible. The activity of the neck extensor muscles was monitored with surface electromyography (EMG) from both sides of the upper trapezius muscle. Surface EMG was recorded using bipolar surface electrodes. Vertical electrooculography was recorded from electrodes placed above and below the left eye. A ground electrode was placed at Fpz in accordance with the international 10–20 system. The impedance of all electrodes was reduced to 5 kΩ. Signals were amplified (×2,000) using a DC amplifier (AN-601G; Nihon Kohden, Tokyo, Japan) for electrooculography and an alternating current (AC) bioelectrical amplifier (MA1000; DIGITEX, Tokyo, Japan) for EMG. Upper trapezius EMG signals were bandpass filtered at 5 to 1,000 Hz and displayed on a digital oscilloscope (DS6612; Iwatsu, Tokyo, Japan).

#### Visual evoked potentials and hemodynamics in the visual cortex

To generate VEP, pattern reversal right-hemi-field visual stimulation was applied to both eyes using a visual stimulator (MTS0410; Medical Try System, Tokyo, Japan). The stimulus was displayed on a 53-cm computer screen placed 64 cm in front of the participant. The visual stimulus (subtending a visual angle of 6° × 6° with a check size of 1° × 1°) was presented to the right of the central fixation point. The visual angle between the left part of the figure and the fixation point was 8°. According to a previous study, the visual information received from this stimulus is almost all projected to the left visual cortex [21,22]. The VEP were recorded using three monopole Ag/AgCl surface electrodes (8-mm diameter); one applied 5 cm above the inion on the midline occipital region, and the other two applied symmetrically, 5 cm lateral to the midline occipital region on the left and right occipital regions. All recording channels were referred to the linked ear lobes. A ground electrode was placed on Cz as defined by the international 10–20 system of electrode placement. Signals from the electrodes were amplified (× 30,000 to 75,000), bandpass-filtered (0.5 to 200 Hz) using the AC bioelectrical amplifier, and sampled at 2,000 Hz using a 16-bit analog-to-digital (A/D) converter (Contec, Osaka, Japan).

**Figure 2:**
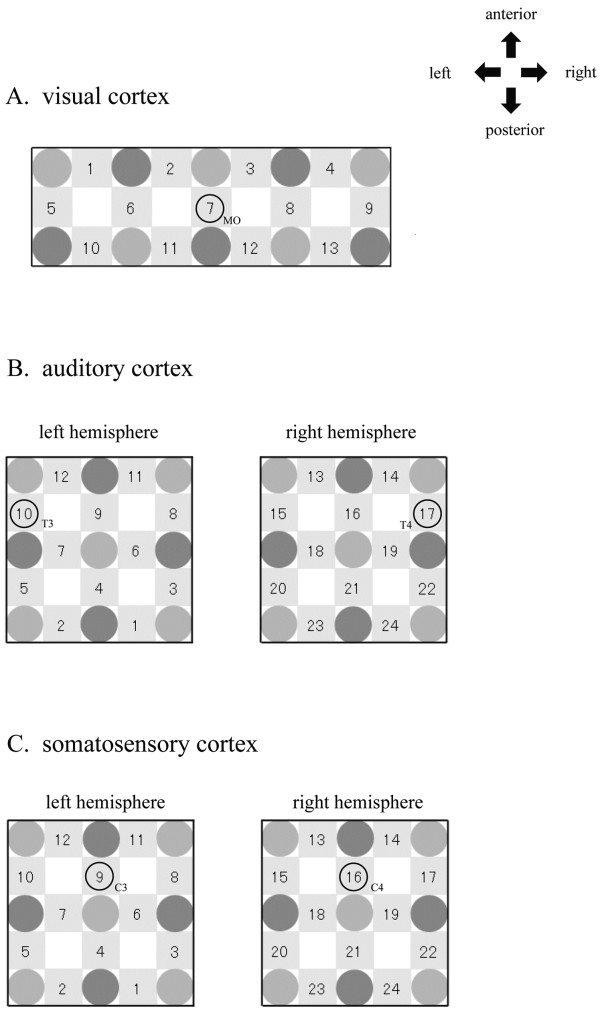
**Schematics indicating the arrangement of optical probes for measurement of oxidative**-**hemoglobin concentration.** Light and dark circles indicate light-emitter and -detector probes, respectively. (**A**) Visual cortex; (**B**) auditory cortex; (**C**) somatosensory cortex.

The hemodynamics in the visual cortex were recorded using a NIRS device (ETG-4000; Hitachi Medical, Tokyo, Japan). A previous study has investigated the concentration of oxidative-hemoglobin (oxy-Hb) at O_1_ and O_2_, as defined by the international 10–20 system during the visual stimulation [23]. We chose to examine this same location in the present study. A probe holder with five light-detector probes and five light-emitter probes was set in a 3 × 12 cm^2^ area including O_1_ and O_2_ positions, and the midline occipital region was positioned at channel 7 in the holder (Figure 2A). Text data of signals captured by the NIRS device were sampled at 10 Hz and sent to a computer.

#### Auditory evoked potentials and hemodynamics in the auditory cortex

To generate AEP, click tones with 0.1-ms duration were generated by an evoked potential system (ER1204; NEC, Tokyo, Japan). The click tones were binaurally delivered via headphones at a rate of 1.0 Hz. The intensity was 50 dB above the predetermined hearing threshold. The evoked potential was recorded from an electrode affixed to the scalp at Cz in accordance with the international 10–20 system, referred to linked ear lobes. A ground electrode was placed on Fpz. Signals from the electrodes were amplified (× 100,000), bandpass-filtered (2 to 2,000 Hz) using the AC bioelectrical amplifier, and sampled at 2,000 Hz using the 16-bit A/D converter.

A previous study has investigated the concentration of oxy-Hb around T3 and T4 positions as defined by the international 10–20 system during the auditory stimulation [17]. We chose to examine this same location in the present study. To measure the hemodynamics in the auditory cortex, two sets of nine probes, consisting of four light-detector probes and five light-emitter probes were fixed over a 6 × 6 cm^2^ area in the left and right temporal cortices. T3 and T4 were positioned at channel 10 and 17 in the probe holders, respectively (Figure 2B).

#### Somatosensory evoked potentials and hemodynamics in the somatosensory cortex

To generate SEP, 0.2-ms square-wave electric pulses were delivered to the median nerve at the left wrist at 120% of the predetermined motor threshold. The stimulation rate was 1.0 Hz. The evoked potential was recorded from electrodes affixed to the scalp, 2 cm posterior from C4 (C4’) and referred to linked ear lobes. A ground electrode was placed on Fpz. Signals from the electrodes were amplified (× 30,000), bandpass-filtered (0.5 to 1,000 Hz) using the AC bioelectrical amplifier, and sampled at 2,000 Hz using the 16-bit A/D converter.

A previous study has investigated the concentration of oxy-Hb around C4 during median nerve electrical stimulation [24]. We chose to examine this same location in the present study. To measure hemodynamics in the somatosensory cortex, the same probe sets as used for the auditory cortex were fixed over the left and right hemispheres. C3 and C4 were positioned at channel 9 and 16 respectively in the probe holders (Figure 2C).

### Procedure

Measurement of evoked potentials and hemodynamics in the three sensory cortices was performed at the same time on three separate days. Prior to the start of measurement, participants contracted and relaxed the shoulder girdle elevator muscles several times, and a deep breath was taken to relax the trapezius muscle. The experimenter verbally instructed the participant to relax the trapezius muscle, and relaxation was confirmed by visual inspection of the EMG signals. Sensory stimuli were delivered while the neck was maintained in a resting position with the chin resting on a stand (resting neck (RN) position) and while neck flexion angle was maintained at 20° with the chin unsupported (flexed neck (FN) position).

**Figure 3:**
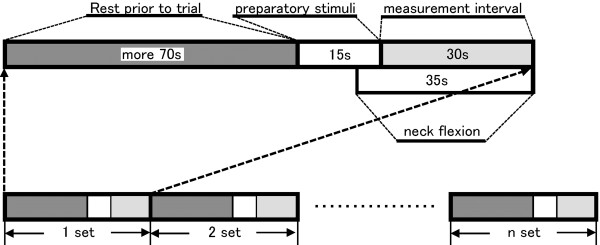
**Schematic indicating the experimental protocol.** Each set consisted of at least 70 s rest, 15 s preparatory sensory stimuli and 30 s sensory stimuli. For the flexed neck target position, the flexed position was adopted after 10 s of preparatory sensory stimulation. The set was repeated as necessary.

The protocol for simultaneous measurement of sensory evoked potentials and oxy-Hb concentration in the sensory cortex is shown in Figure 3. A set consisted of at least 70 s rest, 15 s preparatory sensory stimuli in the RN position and 30 s sensory stimuli at the target neck position (RN or FN). Measurements were acquired during 30 s sensory stimuli at the target neck position. The set was repeated until the average waveform of sensory evoked potentials was obtained. For the FN target position, the data recording at the FN position was started after 15 s from the beginning of preparatory sensory stimulation (10 s of the sensory stimulation, and 5 s needed to move to FN position from RN position). The order of target neck positions was randomly set for each participant. A 3 min rest was taken between conditions. All measurements were completed within 2 h.

### Data analysis

All analyses of evoked potentials were performed using EPLYZER-II (Kissei Comtec, Matsumoto, Japan). Electroencephalography epochs contaminated by blinks or eye movements with amplitudes larger than 100 μV were rejected. Many previous studies have shown that VEP elicited by hemi-field visual stimulation is recorded from the occipital electrode ipsilateral to the visual stimulus side [21,22,25]. Therefore, for the VEP at the right occipital region, 100 epochs from 50 ms before to 250 ms after stimulus onset were obtained. Baseline signal was computed as the average in the 50 ms prior to stimulus onset, and was subtracted from the data before averaging. Latency and peak-to-peak amplitude were analyzed for each of three VEP peaks (N75, P100 and N145) for each individual participant.

For the AEP at Cz, 200 epochs from 20 ms before to 120 ms after stimulus onset were obtained. Baseline signal was computed as the average in the 20 ms prior to stimulus onset, and was subtracted from the data before averaging. Latency and peak-to-peak amplitude were analyzed for each of three AEP peaks (Pa, Nb and Pb) for each individual participant.

For the SEP at C4’, 200 epochs from 20 ms before to 180 ms after stimulus onset were obtained. Baseline signal was computed as the average in the 20 ms prior to stimulus onset, and was subtracted from the data before averaging. Latency and peak-to-peak amplitude were analyzed for each of three SEP peaks (P24, N33, P45) for each individual participant.

According to previous studies [26,27], oxy-Hb concentration is related to the brain blood flow. Baseline oxy-Hb was calculated for the 10 s period prior to preparatory sensory stimulation. The change in oxy-Hb (Δoxy-Hb) during the sensory stimulation was defined as a difference in oxy-Hb from this baseline for each set.

### Statistical analysis

The Shapiro-Wilk test confirmed that all data satisfied the assumption of normality. The latency and amplitude of evoked potentials in each hemisphere were compared across neck positions (RN and FN) using a paired *t*-test. The Δoxy-Hb during the stimulation in the RN position was compared to zero using a one-sample *t*-test. The Δoxy-Hb was compared across neck positions (two levels; RN and FN) and measurement channel using a two-way repeated-measures analysis of variance. When a significant interaction (*P* < 0.05) between these factors was detected, a paired *t*-test was performed separately to assess the difference between postural conditions at each measurement channel. Data are presented as means and SD. The alpha level for statistical significance was set at *P* < 0.05. All statistical analyses were performed using SPSS 14.0J (IBM Japan, Tokyo, Japan).

## Results

### Sensory evoked potentials

**Figure 4:**
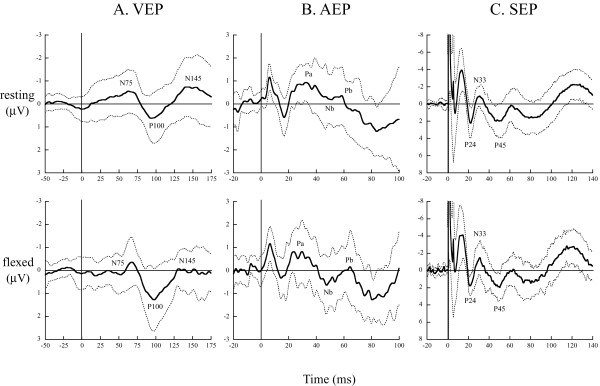
**Grand averaged waveforms of visual** (**A**), **auditory** (**B**) **and somatosensory** (**C**) **evoked potential in resting** (**upper**) **and flexed** (**lower**) **neck positions.** Thick lines are grand averaged waveforms. Dashed lines indicate ±1 SD of a grand averaged waveform. The analyzed components of each evoked potential are labeled. AEP: auditory evoked potentials; SEP: somatosensory evoked potentials; VEP: visual evoked potentials.

**Figure 5:**
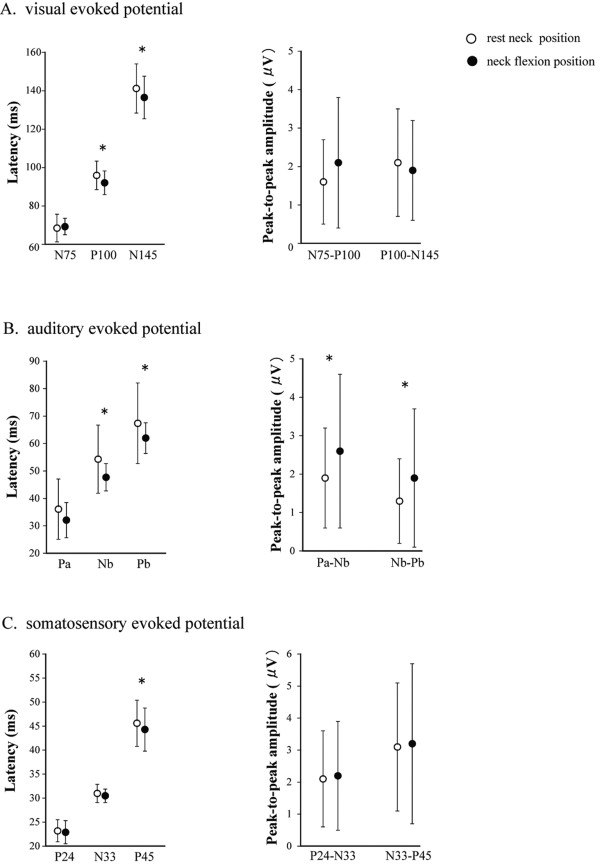
**Latency of each component** (**left panel**) **and peak**-**to**-**peak amplitude of neighboring components** (**right panel**) **of visual** (**A**), **auditory** (**B**) **and somatosensory** (**C**) **evoked potentials in resting** (**open circles**) **and flexed** (**closed circles**) **neck position.** Values represent mean. Error bars represent SD. Asterisk indicate a significant (*P* < 0.05) difference between neck positions.

Figure 4 shows grand averaged waveforms of visual, auditory and somatosensory evoked potential across all participants. All peaks for analysis in the evoked potential were identified. The latency of P100 (*t*_11_ = 3.49, *P* < 0.01) and N145 (*t*_11_ = 2.58, *P* < 0.05) components of VEP, Nb (*t*_11_ = 2.66, *P* < 0.05) and Pb (*t*_11_ = 2.59, *P* < 0.05) components of AEP and the P45 (*t*_11_ = 2.59, *P* < 0.05) component of SEP decreased with neck flexion (Figure 5). The amplitude Pa-Nb (*t*_11_ = 2.59, *P* < 0.05) and Nb-Pb (*t*_11_ = 2.51, *P* < 0.05) components of AEP increased with neck flexion (Figure 5). The amplitude of VEP and SEP components did not change with neck flexion (Figure 5).

### Oxidative-hemoglobin concentration

**Figure 6:**
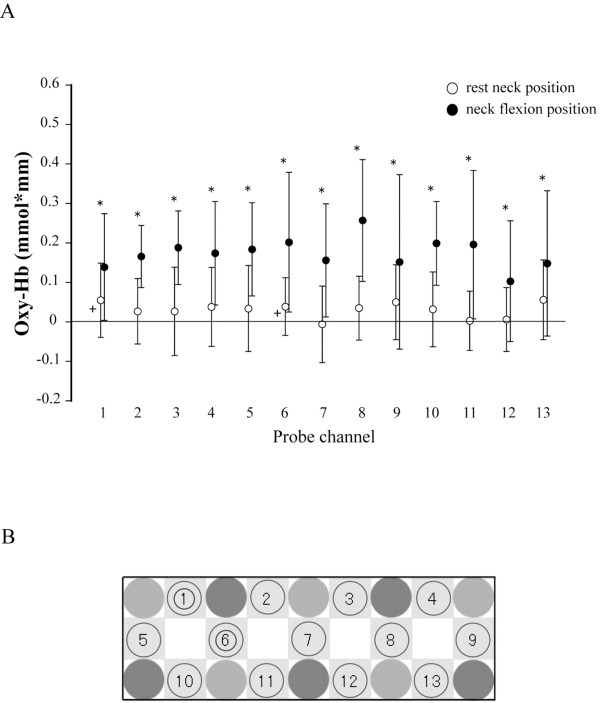
**Oxidative**-**hemoglobin** (**oxy**-**Hb**) **concentration during right hemi**-**field visual stimulation.** (**A**) Mean and standard deviation of oxy-Hb in resting (open circles) and flexed (closed circles) neck positions. Plus signs (+) indicate oxy-Hb concentration significantly greater than zero in the resting neck position. Asterisks indicate a significant difference in oxy-Hb concentration between neck positions. (**B**) A schematic indicating the arrangement of optical probes for measurement of oxy-Hb concentration in the visual cortex. Small circles indicate that oxy-Hb concentration was significantly greater than zero at that channel in the resting neck position. Large circles indicate there was a significant difference in oxy-Hb concentration between neck positions at that channel. oxy-Hb: oxidative-hemoglobin.

During visual stimulation in the RN position, Δoxy-Hb was larger than zero at channels 1 (*t*_11_ = 2.45, *P* < 0.05) and 6 (*t*_11_ = 2.24, *P* < 0.05; Figure 6). A main effect of neck position on Δoxy-Hb during the visual stimulation indicated that Δoxy-Hb was increased with neck flexion (*F*_1,11_ = 22.77, *P* < 0.01; Figure 6). There was no interaction between neck position and measurement channel.

**Figure 7:**
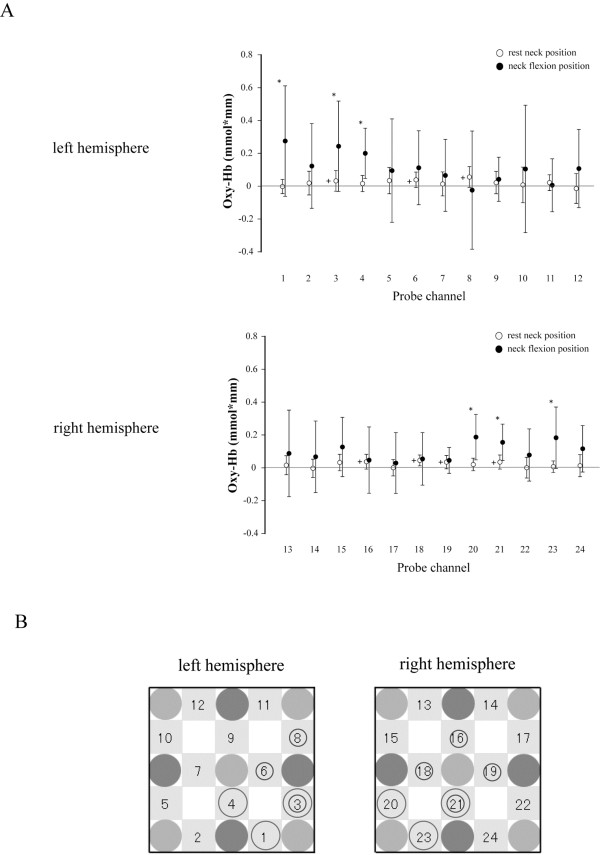
**Oxidative**-**hemoglobin** (**oxy**-**Hb**) **concentration during auditory stimulation.** (**A**) Mean and standard deviation of oxy-Hb in resting (open circles) and flexed (closed circles) neck positions. Plus signs (+) indicate oxy-Hb concentration significantly greater than zero in the resting neck position. Asterisks indicate a significant difference in oxy-Hb concentration between neck positions. (**B**) A schematic indicating the arrangement of optical probes for measurement of oxy-Hb concentration in each hemisphere of the auditory cortex. Small circles indicate that oxy-Hb concentration was significantly greater than zero at that channel in the resting neck position. Large circles indicate there was a significant difference in oxy-Hb concentration between neck positions at that channel. oxy-Hb: oxidative-hemoglobin.

During auditory stimulation in the RN position, Δoxy-Hb was larger than zero at channels 3 (*t*_11_ = 2.23, *P* < 0.05), 6 (*t*_11_ = 2.74, *P* < 0.05) and 8 (*t*_11_ = 2.92, *P* < 0.05) in the left hemisphere and at channels 16 (*t*_11_ = 2.68, *P* < 0.05), 18 (*t*_11_ = 4.61, *P* < 0.05), 19 (*t*_11_ = 2.83, *P* < 0.05) and 21 (*t*_11_ = 2.72, *P* < 0.05) in the right hemisphere (Figure 7). There was an interaction between neck position and measurement channel on Δoxy-Hb during the auditory stimulation (left hemisphere: *F*_11,121_ = 1.92, *P* < 0.01; right hemisphere: *F*_11,121_ = 1.90, *P* < 0.01). Δoxy-Hb was significantly larger in the FN position than in the RN position at channels 1 (*t*_11_ = 2.23, *P* < 0.05), 3 (*t*_11_ = 2.60, *P* < 0.05) and 4 (*t*_11_ = 5.11, *P* <0.01) in the left hemisphere and channels 20 (*t*_11_ = 4.35, *P* < 0.01), 21 (*t*_11_ = 3.92, *P* <0.01) and 23 (*t*_11_ = 3.48, *P* < 0.01) in the right hemisphere (Figure 7).

**Figure 8:**
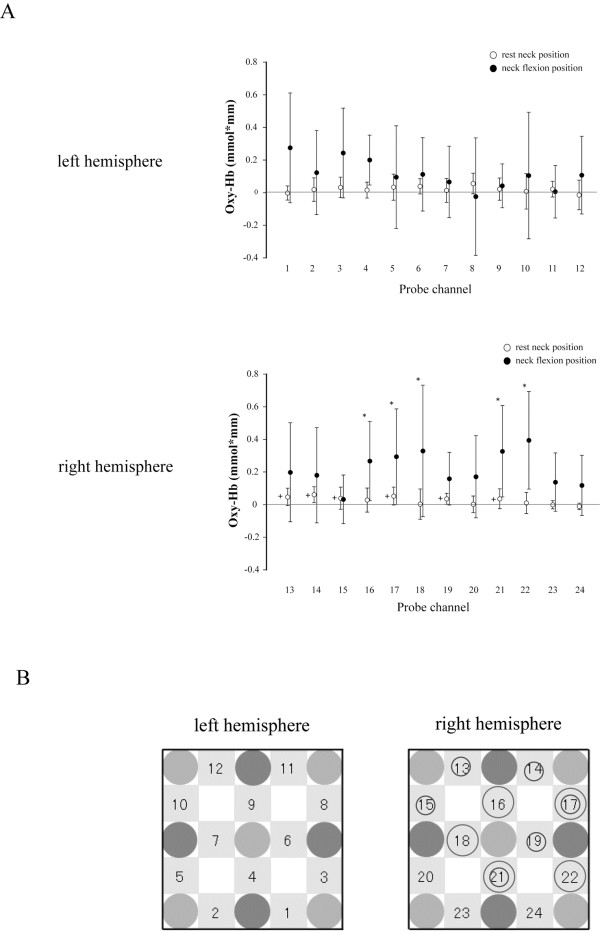
**Oxidative**-**hemoglobin** (**oxy**-**Hb**) **concentration during somatosensory stimulation.** (**A**) Mean and standard deviation of oxy-Hb in resting (open circles) and flexed (closed circles) neck positions. Plus signs (+) indicate oxy-Hb concentration significantly greater than zero in the resting neck position. Asterisks indicate a significant difference in oxy-Hb concentration between neck positions. (**B**) A schematic indicating the arrangement of optical probes for measurement of oxy-Hb concentration in each hemisphere of the somatosensory cortex. Small circles indicate that oxy-Hb concentration was significantly greater than zero at that channel in the resting neck position. Large circles indicate there was a significant difference in oxy-Hb concentration between neck positions at that channel. oxy-Hb: oxidative-hemoglobin.

During somatosensory stimulation at RN position, Δoxy-Hb was significantly larger than zero at channels 13 (*t*_11_ = 3.25, *P* < 0.01), 14 (*t*_11_ = 4.42, *P* < 0.01), 15 (*t*_11_ = 2.34, *P* < 0.05), 17 (*t*_11_ = 3.45, *P* < 0.01), 19 (*t*_11_ = 3.85, *P* < 0.01) and 21 (*t*_11_ = 2.25, *P* < 0.05) in the right hemisphere (Figure 8). There was a significant interaction between neck position and measurement channel on Δoxy-Hb during the somatosensory stimulation (left hemisphere: *F*_11,121_ = 2.62, *P* < 0.01; right hemisphere: *F*_11,121_ = 2.23, *P* < 0.01). Δoxy-Hb was significantly larger in the FN position than in the RN position at channels 16 (*t*_11_ = 3.62, *P* <0.01), 17 (*t*_11_ = 2.98, *P* < 0.05), 18 (*t*_11_ = 2.80, *P* < 0.05), 21 (*t*_11_ = 4.01, *P* < 0.05) and 22 (*t*_11_ = 4.33, *P* < 0.05) in the right hemisphere (Figure 8).

## Discussion

FBBF indicated that the sensory cortex was locally activated during sensory stimulation when the neck was in a resting position. This is consistent with previous studies reporting an increase in FBBF in the activated sensory cortex, according to the sensory stimulation [17-19,23,24]. The results of the present study extend these findings to show that the sensory evoked potential and FBBF change with neck flexion. The latencies of VEP (P100 and N145 components), AEP (Nb and Pb components) and SEP (P45 component) were shorter, and the amplitude of AEP (Pa-Nb and Nb-Pb components) was larger, when the neck was flexed. The increase in FBBF associated with neck flexion was not observed in the entire cortex but only in the cortices activated by stimulation.

Visual information received by the retina reaches the primary visual cortex via the lateral geniculate body in the thalamus [25,28]. According to dipole source analysis, N75 and P100 components of VEP originate in the striate cortex [25,29-32], and the N145 component originates in the striate cortex [29,32] and/or the extrastriate cortex [30]. Auditory signals reach the primary auditory cortex (Brodman area 41 and 42) with a latency of 10 to 12 ms [33]. It has been suggested that the middle-latency components of the AEP (Na: 16 to 20 ms, Pa: 27 to 32 ms, Nb: 37 to 43 ms) originate in the primary and secondary auditory cortices [34,35]. The N20/P20 component of the SEP originates from Brodman’s area 3b [36-39]. The P25 and N30/P30 components of the SEP originate in the primary somatosensory cortex [36,39-42] and the P45 component originates is in the secondary somatosensory area, including Brodman’s area 5 [43]. It is clear from the present results that the cortical areas in the neural pathways related to visual, auditory and somatosensory information processing were activated with neck flexion.

The increase in FBBF in the visual cortex with neck flexion was observed in both the left and right visual cortices. The left visual cortex receives visual information from right visual field, whereas the right visual cortex does not directly receive visual information. The increase in FBBF in the auditory cortex was also bilateral, but an increase in FBBF was only observed in the right somatosensory cortex. The density of fiber connections between the hemispheres is higher in the visual cortex than in the somatosensory cortex [44]. The results of the present study suggest that activation in sensory cortices with neck flexion reflects sensory information processing in the cerebral cortex and the reciprocal inter-hemispheric connections.

Mechanisms of selective activation in sensory cortices cannot be determined from the present data; however, we can speculate on contributing factors. The thalamus is one of the main centers of brain activation and plays an important role as a relay portion for sensory information. The sensory relay pathway to the cortex in the thalamus may be affected by the ascending brain activation originating from the brainstem reticular formation and/or descending brain activation originating from the frontal lobe, which includes attention [6-10]. In addition, functional coupling between the frontal lobe and the sensory cortex may lead to the selective activation in the related sensory cortex [45,46]. If the thalamus played a key role, it would be expected that the early component of the sensory evoked potential should be activated while maintaining the neck flexion. However, we observed no activation or shortening of latency for the early components of evoked responses, and we therefore presume that the effect of neck flexion occurs within the cortex at a location other than the thalamus. However, further research is required to determine the mechanism of selective activation in sensory cortices associated with neck flexion.

Facilitation of visual, auditory and somatosensory information processing and the enhancement of activity in related sensory cortices accompanied by maintaining the neck flexion position must be useful for perceiving the surrounding environment, the location of the whole body in the environment and the position of each segment of the body. In previous studies, the shortening of saccadic reaction time associated with the neck flexion position was observed after the saccadic training with maintenance of neck flexion [3,20]. The present results could be effectively applied to older participants or be used in clinical investigations for patients with sensory perception impairments.

## Conclusions

We have demonstrated that visual, auditory and somatosensory pathways are activated with neck flexion; however, the cerebral activation differed between the pathways. The differences in selective activation between the sensory cortices reflect the modalities in sensory projection to the cerebral cortex and inter-hemispheric connections.

## Abbreviations

AC: Alternating current; A/D: Analog-to-digital; AEP: Auditory evoked potentials; EMG: Electromyography; FBBF: Focal brain blood flow; fMRI: Functional magnetic resonance imaging; FN: Flexed neck; NIRS: Near-infrared spectroscopy; oxy-Hb: Oxidative-hemoglobin; RN: Resting neck; SD: Standard deviation; SEP: Somatosensory evoked potentials; VEP: Visual evoked potentials.

## Competing interests

The authors have no competing interests to disclose.

## Authors' contributions

Contribution of each author is as follows: KF presented all the idea of this study, planed the method, directed the experiments and interpreted the results. Most sentences in Introduction and Discussion including Conclusion were written by KF, KK and AM. KK, NK, AM and MI contributed to the experiments, data analyses and wrote the sections of Experimental procedures and Results. They also discussed about all part of manuscript with KF. All authors read and approved the final manuscript.
